# Comparative Evaluation of the BD Phoenix Yeast ID Panel and Remel RapID Yeast Plus System for Yeast Identification

**DOI:** 10.1155/2016/4094932

**Published:** 2016-04-14

**Authors:** Michelle L. Grant, Shobha Parajuli, Raquel Deleon-Gonsalves, Raghava Potula, Allan L. Truant

**Affiliations:** ^1^Departments of Pathology and Laboratory Medicine, Temple University Hospital and School of Medicine, Philadelphia, PA 19140, USA; ^2^Clinical Microbiology, Immunology and Virology Laboratories, Temple University Hospital and School of Medicine, Philadelphia, PA 19140, USA; ^3^Microbiology and Immunology, Temple University Hospital and School of Medicine, Philadelphia, PA 19140, USA; ^4^Internal Medicine, Temple University Hospital and School of Medicine, Philadelphia, PA 19140, USA

## Abstract

Becton Dickinson Phoenix Yeast ID Panel was compared to the Remel RapID Yeast Plus System using 150 recent clinical yeast isolates and the API 20C AUX system to resolve discrepant results. The concordance rate between the Yeast ID Panel and the RapID Yeast Plus System (without arbitration) was 93.3% with 97.3% (146/150) and 95.3% (143/150) of the isolates correctly identified by the Becton Dickinson Phoenix and the Remel RapID, respectively, with arbitration.

## 1. Introduction

Epidemiology of invasive fungal infections is evolving. Fungemia is now the fourth leading cause of bloodstream infections [1–3] with an increasing number of* Candida* yeast infections from non-*albicans* species [4–6]. These infections may be associated with high morbidity and mortality rates [7–10]; however, enhanced preventive measures, earlier detection, and implementation of proper treatment [4, 11] have slowly improved disease outcomes. Nevertheless, the increasing development of candidemia and cryptococcal infection resistance to fluconazoles and other azoles [1, 2, 6, 11–14] underscores the urgent need for accurate and rapid detection methods. New identification platforms like matrix-assisted laser desorption/ionization time of flight mass spectrometry (MALDI-ToF MS) and DNA sequence based methods are promising solutions [15], but their high cost and limited organism databases have kept traditional commercial phenotypic tests as the main identification method for yeast and in most clinical laboratories. These tests range from labor-intensive manual panels to newer automated detection systems.

The purpose of this study was to compare the performance of the automated Becton Dickinson (BD, Sparks, MD) Phoenix Yeast ID Panel (Phoenix) to a manual Remel RapID Yeast Plus System (RYP, Remel, Thermo Fisher Scientific, Lenexa, KS). The BD Yeast ID Panel, first introduced in 2011, is for use on the Phoenix Microbiology System. The BD Yeast ID Panels are self-inoculating molded polystyrene trays containing 3 control wells (1 negative fluorescent control and 2 positive fluorescent control) and 47 wells with dried biochemical substrates that use traditional qualitative microbiology methods such as fermentation, oxidation, degradation, and hydrolysis in combination with chromogenic and fluorogenic substrates along with carbon sources for identification. Previous studies demonstrated an accuracy of 94.4% and 84% [16, 17]. The RYP is a qualitative micromethod that uses 18 conventional and chromogenic substrates for identification. In previously published studies, RYP has demonstrated accuracies within 77–97% [18–22].

## 2. Methods

150 yeasts and isolates encompassing 11 species were tested. Isolates were obtained from clinical samples (blood, urine, tissue, CSF, pleural fluid, and bronchial aspirate) collected from patients at Temple University Hospital, in Philadelphia, PA, over a 6-month period. Specimens were subcultured to Sabouraud-Emmons dextrose agar plates (Remel, Thermo Fisher Scientific, Lenexa, KS) and incubated at 30°C for 24 or 48 hours as required by Phoenix and RYP, respectively. All study isolates were simultaneously tested with the BD Phoenix and RYP systems, according to the instructions of each ID panel. While the BD Phoenix result was automated, the RYP system required reaction interpretation based on color changes that were used to obtain a numeric code for a computer database of yeast. This analysis was performed by 2 individuals simultaneously. Both systems produced a result with a confidence indicator. A result was accepted for the Phoenix if the confidence value was >90%, while results listed as implicit, satisfactory, or adequate were accepted for RYP. If an unacceptable result was obtained for either test, both tests were repeated and the results from the additional run were used if they met the criteria. If the Phoenix and RYP results were in agreement, the isolate was considered correctly identified. Where Phoenix and RYP systems disagreed, the isolate was further analyzed by the API 20C AUX system, which utilized corn meal morphology and colorimetric tests for conventional assimilation substrates, actidione resistance, and phenoloxidase production. The identification made by two of the three systems was considered correct.

## 3. Results

Table 1 shows species identified by the two systems. The four most common species were* C. albicans* (27%),* C. glabrata* (26%),* C. tropicalis *(19%), and* C. parapsilosis* complex (13%). Overall, the Phoenix and RYP agreed for 140 of the 150 isolates (93.3%). Of the 10 discrepancies, the reference test (API) agreed with the Phoenix for 6 isolates and the RYP for 3 isolates. These results were not statistically significant (*Z*-score 0.948; approximate* P *= 0.1736) and are similar to a recently published study by Gayibova et al. who demonstrated 88% concordance with API ID 32C AUX system [23]. Neither study utilized molecular or proteomic identification of the yeast isolates; therefore, determinative sensitivity, specificity, and positive and negative predictive values were not appropriate for this evaluation.

Phoenix incorrectly identified 3 frequently encountered clinical yeast isolates (2* Candida* spp. and 1* Cryptococcus* sp., Table 2). Two isolates of* C. glabrata *(accuracy for* C. glabrata* 94.9%) and 1 isolate of* C. tropicalis *(accuracy for* C. tropicalis* 96.4%) were misidentified and a* Cryptococcus laurentii* was identified by the Phoenix as a* Trichosporon asahii*. Two* C. glabrata* were misidentified by the Phoenix as* C. firmetaria*, and one* C. tropicalis* was misidentified as a* C. pelliculosa*. Repeat testing of the* C. glabrata* isolates (by the Phoenix) was identified correctly while* C. tropicalis *remained incorrect. Previous studies demonstrated 100% accuracy when identifying* C. glabrata* but only 93.3–100% with* C. tropicalis* [16, 17]. The initial misidentification of* C. glabrata *by the Phoenix may be of some concern since it is the second most common cause of fungemia in the USA [4] whose susceptibility to commonly used therapies fluconazole and amphotericin B is decreasing [3].

In this evaluation, the RYP misidentified 3 yeast isolates into 7 yeast organisms (Table 2).* Candida parapsilosis* was identified by the RYP into five yeast isolates representing four* Candida* spp. (*C. guilliermondii, C. lambica, C. lusitaniae, and C. zeylanoides*) resulting in 73.7% accuracy for the identification of* C. parapsilosis* using this system. After repeat testing, the misidentified isolates were correctly identified in 3 of the 5 isolates. This rate of misidentification for* C. parapsilosis* was not reported previously [18–22]. The remaining misidentifications by the RYP consisted of a* Cryptococcus laurentii* identified as a* Trichosporon beigelii* and a* Trichosporon asahii* identified as a* Trichosporon beigelii*.

Both the Phoenix and the RYP systems were unable to identify one isolate of* Cryptococcus laurentii* but instead speciated it into a* Trichosporon* species. As per manufacture instructions, the BD Phoenix requires growth on blood agar to identify* C. laurentii* and no recommendations were made for RYP. Therefore, the isolate was subcultured on blood agar and retested in parallel. Both the Phoenix and the RYP correctly identified the isolates that were grown on blood agar. Although* C. laurentii* is known as a biopesticide, clinical infections from it have been reported most commonly in immunosuppressed patients [24]. Therefore, the use of media not recommended by the manufacturer could result in erroneous identification of this unusual organism. Won et al. tested* C. laurentii *isolates with the Phoenix using both media (Sabouraud and blood agars) and the Phoenix was able to identify each one correctly [16]. The significance of the media for certain isolates of* C. laurentii* needs to be further elucidated.

## 4. Conclusion 

In summary, the automated Phoenix is comparable to the manual RYP system with a concordance rate of 93.3%. Posteraro and Won demonstrated accuracies for the Phoenix of 94.4% and 84% (using 250 and 351 yeast isolates, resp.). These combined results support the reliability of the Phoenix system. However, it should be noted that if the yeast isolates from the previous two studies were divided into common yeast species and the rare yeast species the accuracy of the Phoenix varied significantly: 98–98.2% for commonly encountered species and 70–76% for rarely encountered species [16, 17]. This suggests that larger databases may be needed to improve discrimination among rarely encountered yeast species. Previous studies also demonstrated quicker turnaround times when compared to other manual and automated systems. The Phoenix required 4–15 hours for identification after growth for 24 hours [17], while the manual RYP and API systems require a minimum of 52 hours (RYP) and 96 hours (API). Therefore, the Phoenix system appears to be a comparable yeast identification system while requiring less incubation time, ultimately allowing for quick and reliable results.

## Figures and Tables

**Table 1:** Identification results obtained with the Phoenix and RYP systems for 150 clinical yeast isolates^a^.

Species (number tested)	Number (%) of isolates with indicated result			
	Identified		Misidentification	
	Phoenix	RYP	Phoenix	RYP
*Candida albicans *(41)	41	41	0	0
*Candida glabrata* (39)	37	39	2	0
*Candida tropicalis* (28)	27	28	1	0
*Candida parapsilosis* (19)	19	14	0	5
*Candida krusei* (8)	8	8	0	0
*Cryptococcus neoformans *(8)	8	8	0	0
*Candida lipolytica *(1)	1	1	0	0
*Cryptococcus albidus* (1)	1	1	0	0
*Cryptococcus laurentii *(1)	0	0	1	1
*Trichosporon asahii *(1)	1	0	0	1

Total (150)	146 (97.3)	143 (95.3)	4 (2.7)	7 (4.7)

^a^After arbitration using API 20C AUX.

**Table 2:** Discrepant identifications by Phoenix and RYP systems for 10 yeast isolates.

Results for indicated identification method (number misidentified)		
Reference^a^	Phoenix	RYP
*Candida glabrata*	*Candida fermetaria* (2)	

*Cryptococcus laurentii*	*Trichosporon asahii *(1)	*Trichosporon beigelii* (1)

*Candida parapsilosis*		*Candida guilliermondii* (1) *Candida lambica* (2) *Candida lusitaniae *(1) * Candida zeylanoides *(1)

*Candida tropicalis*	*Candida pelliculosa *(1)	

*Trichosporon asahii*		*Trichosporon beigelii *(1)

^a^Identifications were confirmed by using API 20C AUX.

## References

[1] Zilberberg M. D., Shorr A. F., Kollef M. H. (2008). Growth and geographic variation in hospitalizations with resistant infections, United States, 2000–2005. *Emerging Infectious Diseases*.

[2] Wisplinghoff H., Bischoff T., Tallent S. M., Seifert H., Wenzel R. P., Edmond M. B. (2004). Nosocomial bloodstream infections in US hospitals: analysis of 24,179 cases from a prospective nationwide surveillance study. *Clinical Infectious Diseases*.

[3] Pfaller M. A., Diekema D. J. (2010). Epidemiology of invasive mycoses in North America. *Critical Reviews in Microbiology*.

[4] Horn D. L., Neofytos D., Anaissie E. J. (2009). Epidemiology and outcomes of candidemia in 2019 patients: data from the prospective antifungal therapy alliance registry. *Clinical Infectious Diseases*.

[5] Hope W., Morton A., Eisen D. P. (2002). Increase in prevalence of nosocomial non-*Candida albicans* candidaemia and the association of *Candida krusei* with fluconazole use. *Journal of Hospital Infection*.

[6] Arendrup M. C., Dzajic E., Jensen R. H. (2013). Epidemiological changes with potential implication for antifungal prescription recommendations for fungaemia: data from a nationwide fungaemia surveillance programme. *Clinical Microbiology and Infection*.

[7] Morgan J., Meltzer M. I., Plikaytis B. D. (2005). Excess mortality, hospital stay, and cost due to candidemia: a case-control study using data from population-based candidemia surveillance. *Infection Control and Hospital Epidemiology*.

[8] Morrell M., Fraser V. J., Kollef M. H. (2005). Delaying the empiric treatment of *Candida* bloodstream infection until positive blood culture results are obtained: a potential risk factor for hospital mortality. *Antimicrobial Agents and Chemotherapy*.

[9] Pappas P. G., Rex J. H., Lee J. (2003). A prospective observational study of candidemia: epidemiology, therapy, and influences on mortality in hospitalized adult and pediatric patients. *Clinical Infectious Diseases*.

[10] Wey S. B., Mori M., Pfaller M. A., Woolson R. F., Wenzel R. P. (1988). Hospital-acquired candidemia. The attributable mortality and excess length of stay. *Archives of Internal Medicine*.

[11] Pfaller M., Neofytos D., Diekema D. (2012). Epidemiology and outcomes of candidemia in 3648 patients: data from the prospective antifungal therapy (PATH Alliance®) registry, 2004–2008. *Diagnostic Microbiology and Infectious Disease*.

[12] Garey K. W., Rege M., Pai M. P. (2006). Time to initiation of fluconazole therapy impacts mortality in patients with candidemia: a multi-institutional study. *Clinical Infectious Diseases*.

[13] Datta K., Rhee P., Byrnes E. (2013). Isavuconazole activity against *Aspergillus lentulus*, *Neosartorya udagawae*, and *Cryptococcus gattii*, emerging fungal pathogens with reduced azole susceptibility. *Journal of Clinical Microbiology*.

[14] Almirante B., Rodríguez D., Park B. J. (2005). Epidemiology and predictors of mortality in cases of Candida bloodstream infection: results from population-based surveillance, Barcelona, Spain, from 2002 to 2003. *Journal of Clinical Microbiology*.

[15] Griffin A. T., Hanson K. E. (2014). Update on fungal diagnostics. *Current Infectious Disease Reports*.

[16] Won E. J., Shin J. H., Kim M.-N. (2014). Evaluation of the BD Phoenix system for identification of a wide spectrum of clinically important yeast species: a comparison with Vitek 2-YST. *Diagnostic Microbiology and Infectious Disease*.

[17] Posteraro B., Ruggeri A., De Carolis E. (2013). Comparative evaluation of BD phoenix and Vitek 2 systems for species identification of common and uncommon pathogenic yeasts. *Journal of Clinical Microbiology*.

[18] Wadlin J. K., Hanko G., Stewart R., Pape J., Nachamkin I. (1999). Comparison of three commercial systems for identification of yeasts commonly isolated in the clinical microbiology laboratory. *Journal of Clinical Microbiology*.

[19] Verweij P. E., Breuker I. M., Rijs A. J. M. M., Meis J. F. G. M. (1999). Comparative study of seven commercial yeast identification systems. *Journal of Clinical Pathology*.

[20] Sanguinetti M., Porta R., Sali M. (2007). Evaluation of VITEK 2 and RapID Yeast plus systems for yeast species identification: experience at a large clinical microbiology laboratory. *Journal of Clinical Microbiology*.

[21] Moghaddas J., Truant A. L., Jordan C., Buckley H. R. (1999). Evaluation of the RapID Yeast Plus System for the identification of yeast. *Diagnostic Microbiology and Infectious Disease*.

[22] Heelan J. S., Sotomayor E., Coon K., D'Arezzo J. B. (1998). Comparison of the rapid yeast plus panel with the API20C yeast system for identification of clinically significant isolates of *Candida* species. *Journal of Clinical Microbiology*.

[23] Gayibova U., Dalyan Cilo B., Agca H., Ener B. (2014). Comparison of Phoenix Yeast ID Panel and API(R) ID 32C commercial systems for the identification of *Candida* species isolated from clinical samples. *Mikrobiyoloji Bulteni*.

[24] Shankar E. M., Kumarasamy N., Bella D. (2006). Pneumonia and pleural effusion due to *Cryptococcus laurentii* in a clinically proven case of AIDS. *Canadian Respiratory Journal*.

